# Independent Component Analysis for Source Localization of EEG Sleep Spindle Components

**DOI:** 10.1155/2010/329436

**Published:** 2010-03-29

**Authors:** Erricos M. Ventouras, Periklis Y. Ktonas, Hara Tsekou, Thomas Paparrigopoulos, Ioannis Kalatzis, Constantin R. Soldatos

**Affiliations:** ^1^Department of Medical Instrumentation Technology, Technological Educational Institution of Athens, Ag. Spyridonos Street, Egaleo,12210 Athens, Greece; ^2^Sleep Research Unit, Eginition Hospital, Department of Psychiatry, University of Athens, 74 Vas.Sophias Avenue, 11528 Athens, Greece

## Abstract

Sleep spindles are bursts of sleep electroencephalogram (EEG) quasirhythmic activity within the frequency band of 11–16 Hz, characterized by progressively increasing, then gradually decreasing amplitude. The purpose of the present study was to process sleep spindles with Independent Component Analysis (ICA) in order to investigate the possibility of extracting, through visual analysis of the spindle EEG and visual selection of Independent Components (ICs), spindle “components” (SCs) corresponding to separate EEG activity patterns during a spindle, and to investigate the intracranial current sources underlying these SCs. Current source analysis using Low-Resolution Brain Electromagnetic Tomography (LORETA) was applied to the original and the ICA-reconstructed EEGs. Results indicated that SCs can be extracted by reconstructing the EEG through back-projection of separate groups of ICs, based on a temporal and spectral analysis of ICs. The intracranial current sources related to the SCs were found to be spatially stable during the time evolution of the sleep spindles.

## 1. Introduction

Sleep spindles are characteristic transient oscillations that appear on the electroencephalogram (EEG) during nonrapid eye movement (non-REM) sleep. They are characterized by progressively increasing, then gradually decreasing waveforms with frequencies ranging from 11 to 16 Hz. Sleep spindles characterize sleep onset, being one of the defining EEG waveforms of stage 2 sleep. They are affected by medication, aging, and brain pathology and may be involved in learning processes [1]. Analyses of scalp-recorded sleep spindles have demonstrated topographic distinction between two sleep spindle classes: “slow” spindles, with spectral peak frequency at around 12 Hz, and “fast” spindles, with spectral peak frequency at around 14 Hz. Slow spindles are more pronounced over frontal scalp electrodes, while fast spindles exhibit mainly parietal and central scalp distribution [2–5]. 

Independent Component Analysis (ICA) is a statistical technique used for solving the Blind Source or Signal Separation (BSS) problem [6, 7]. Suppose that data measured in an experiment are expressed through an *n-*dimensional vector **y**(*k*) = [*y*_1_(*k*),…,*y*_*n*_(*k*)]^*T*^, for  *k* = 1,…, *N*_sample_, where *N*_sample_ is the number of measured data time samples. The BSS problem relates to recovering unknown “source” signals *s*_1_(*k*),…, *s*_*m*_(*k*) from their mixtures, that is, the measured data, without prior knowledge about the mixing mechanism producing the measured data. Sources and measured data are related through

(1) $$\;\text{y}\left(k\right)=\text{Ms}\left(k\right),\;k=1,\ldots ,N_{\text{sample}},$$

where **M** = [**m**_1_,…, **m**_*m*_] is the unknown “mixing” matrix. It should be noted that, in the BSS context, the term “sources” does not refer to physical sources of the measured data, but to the mathematical entities that could satisfy (1). 

The source signal, for each ICA source *s*_*j*_  (*j* = 1,…, *m*), is assumed to represent a random variable, whose sample values are *s*_*j*_(1),…, *s*_*j*_(*N*_sample_). The source signal random variables would be statistically independent if their joint probability density function (pdf) *f*(*s*_1_ ⋯ *s*_*m*_) could be factored as follows:

(2) $$\;f\left(s_{1},\ldots ,s_{m}\right)=f_{1}\left(s_{1}\right)f_{2}\left(s_{2}\right)\cdots f_{m}\left(s_{m}\right),$$

where *f*_*j*_(*s*_*j*_) denotes the marginal pdf of *s*_*j*_.

ICA tries to estimate sources as linear projections of the measured data, based on the criterion that the resulting source time courses *s*_*j*_(1),…, *s*_*j*_(*N*_sample_)  (*j* = 1,…, *m*), that is, the ICA source signal random variables, should be as statistically independent as possible [6]. Each estimated source is called an independent component (IC). In a more general aspect, in the case of time-series data, it is assumed that each ICA source is generated by a random process, which is independent of the random processes generating the other sources.

The solution is in the form

(3) $${\text{s}}_{\text{est}}\left(k\right)=\text{Wy}\left(k\right),\;k=1,\ldots ,N_{\text{sample}},$$

where **W** is called the “unmixing” matrix. ICs can be determined up to a multiplicative sign [8], which may vary across ICs. Due to this indeterminacy, ICs cannot be used for directly extracting quantitative measures from their values. Rather their characteristics, such as their waveform morphology, indicate that they represent original independent sources. Quantitative measures have to be extracted from “reconstructed” data, which are reprojections of ICs, through the mixing matrix [9].

 When ICA is applied to electrical signals (mixtures) recorded from the human body, it would be interesting to investigate whether the current source regions of the recorded signals, inside the human body, remain spatially fixed for the duration of the recorded data. This characteristic of current source regions would have special importance for EEG data. It might be expressed through the qualification of “spatial stationarity” for the current sources, meaning that the EEG, reconstructed from a set of ICs, is generated by current sources which have stable locations for the duration of the recorded data [9, 10]. The spatial stationarity characteristic would be desirable because, if ICA could help in finding spatially stable intracranial current sources, it might shed light to the localization of various brain processes. ICA has been extensively used in EEG signal processing applications, including noise elimination, component extraction of Event-Related Potentials (ERPs), and single-trial ERP analysis [10, 11].

In the case of sleep spindles, in the time frame of a single spindle detected by a human scorer, there often seems to exist separate spindle “components” (SCs), with different frequency spectra and/or electrode distribution. Differentiating SCs in the context of investigating related intracranial current sources seems challenging, since SCs might overlap in space and time. In previous work, the extraction of such SCs has been investigated by applying ICA to sleep spindle EEG [12]. 

Techniques used for solving the inverse problem in order to detect intracranial current sources of scalp-recorded EEG, which assume a distributed current source model, have been extensively used in recent years [13]. In these models, extended brain areas are represented by a three-dimensional grid of solution points. Each point is a possible location of a current source. This approach does not pose restrictions on the number and focality of sources to be computed. It is suitable when there are no specific indications about source locations and extent. On the other hand, the number of source points can be much larger than the number of measurement points on the scalp surface. This makes the inverse problem a heavily undetermined one, resulting in source distributions that are rather diffuse and extended. Among the techniques assuming a distributed current source model, Low-Resolution Brain Electromagnetic Tomography (LORETA) is one of the most extensively used [14, 15]. LORETA solves the inverse problem by assuming that the orientations and strengths of neighboring neuronal sources are correlated, because neuronal activity in neighboring patches of cortex is expected to be correlated. Mathematically, this assumption is implemented by finding the “smoothest” of all possible source density distributions. The LORETA version presented by Pascual-Marqui et al. in [15] considered a three-shell spherical head model that was registered to the Talairach human brain atlas [16]. The solution space was restricted to the cortical gray matter and the hippocampus. 

Based on recent research applying LORETA to visually detected sleep spindles, there exist indications that the difference in the frequency and topography of the two sleep spindle classes reflects electrical activity related to spindle oscillations at two broadly distinct cortical areas: fast spindling source activity found posteriorly and slow spindling source activity found anteriorly [17]. Concordant LORETA results were obtained in the study of Durka et al. [18], using multichannel matching pursuit as a preprocessing step for automatic detection and parameterization of sleep spindles. Furthermore, indications for the existence of different and independent cortical circuits generating the two classes of spindles have been provided by a study of electrocorticographic (EcoG) potentials from electrodes located in the prefrontal cortex [19]. On the other hand, the two frequency classes have also been attributed to a single mechanism, the duration of hyperpolarization-rebound sequence in thalamocortical neurons. Accordingly, long hyperpolarizations generate slower EEG frequencies, while short hyperpolarizations create faster EEG frequencies [20]. Indications for considering both slow and fast spindle activity as a single event in global thalamocortical coherence have been provided by a recent magnetoencephalographic (MEG) source localization study [21]. Also, the neuronal transition probability model proposed by Merica and Fortune [22] invokes oscillatory modes of different frequencies existing simultaneously in one fixed-size neuronal population source. The hypothesis that there is only one kind of sleep spindle and an anterior peak of alpha EEG activity during non-REM sleep has also been supported [1, 23, 24]. Therefore, the question as to whether there exist one or two functionally separate sleep spindle generators, related to the fast and slow spindle classes, should be considered open.

The aim of the present study is to investigate the application of ICA to sleep spindle EEG, in order to enable the localization of intracranial current sources for SCs using LORETA. The methodology that is proposed may contribute to the on-going research concerning the existence of distinct intracranial current sources for the slow and fast spindle classes. Furthermore, an investigation is carried concerning whether SCs are generated by intracranial current sources with stable locations, that is, whether the current sources for SCs possess a “spatial stationarity” characteristic for the whole duration of the sleep spindle.

## 2. Material and Methods

### 2.1. Sleep EEG Recording Procedure and Preprocessing

A healthy 27-year-old male subject slept for one night in the Sleep Research Unit of the Department of Psychiatry at the University of Athens Medical School. Informed consent was obtained from the subject, and the study protocol was approved as appropriate. The all-night polysomnogram was recorded digitally utilizing a Micromed/BrainQuick system. The EEG was recorded with 21 electrodes (referential montage, reference at G2), at positions F8, T4, T6, Fp2, F4, C4, P4, O2, Fpz, Fz, Cz, Pz, Oz, Fp1, F3, C3, P3, O1, F7, T3, T5, of the International 10/20 EEG electrode positioning system, with sampling frequency 512 Hz. Visual evaluation of the sleep record was carried out by an experienced polysomnographer, utilizing standard procedures [25], and was verified by a second one. The sleep EEG record was divided into stages and sleep spindles were visually detected from sleep stage 2, because sleep spindles are more prevalent in this sleep stage [1]. The sleep spindles were filtered using a 128th-order finite impulse response (FIR) bandpass filter, with 3 dB cut-off frequencies at 10.5 and 16 Hz, using the software package Matlab (The MathWorks Inc.).

### 2.2. Computation of Independent Components

**Figure 1:**
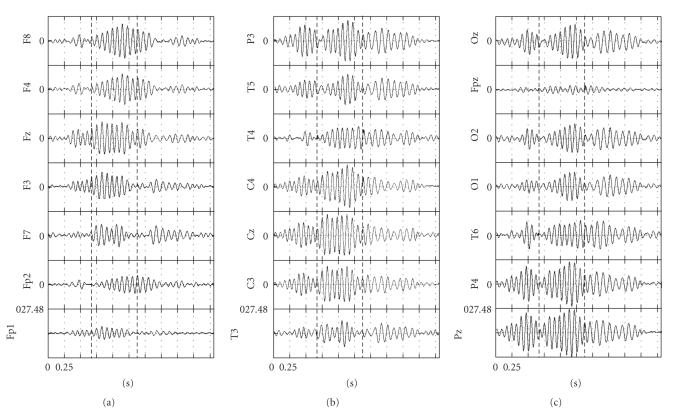
Bandpass-filtered 21-channel EEG sleep spindle recording. In each electrode recording plot the first (second) vertical dashed line indicates the time point of transition from part A (B)of the original single-spindle timeframe topart B (C). Potentials of EEG are in microvolts.

ICA was applied on the original bandpass-filtered EEG data (Figure 1), using the FastICA algorithm [26]. In order to check whether the number of time samples available was sufficient for providing stable Independent Components (ICs) [9], the bandpass-filtered EEG data were upsampled 2, 4, 8, and 16 times. For all sampling rates, no differences were found between the computed ICs. 

The ICs, which were produced when a 21 × 21 unmixing matrix was computed, were composed of short-duration wavelets, with no apparent spindle-like activity and/or correspondence to the EEG spindle activity [27]. It should be noted that this was not due to algorithmic reasons related to the FastICA algorithm, since the same picture emerged for ICs when either the infomax [28] or JADE (Joint Approximate Diagonalization of Eigen-matrices) [29] algorithms were applied, using the EEGLAB package [30]. In addition, this was not due to the number of time samples available, since the phenomenon was present even when the signal was highly upsampled (see above). In order to overcome the problem described above, dimensionality reduction was applied. The original data were first “centered”; that is, the data vector was transformed to a zero-mean variable. Then, dimensionality reduction was applied, in which a subset of the eigenvalues of the covariance matrix of the “centered” data was retained [7, 27, 31]. The dimensionality reduction resulted in the computation of *n*_red_(<21) ICs. *n*_red_ was selected as the number of dominant eigenvalues which accounted for 99% of the total variance of the bandpass-filtered EEG signal. Consequently, an *n*_red_ × 21 unmixing matrix was computed instead of a 21 × 21 matrix. This procedure has been previously applied in ICA studies, where the dimensionality of the problem was reduced, by computing a sub-set of the rows of the unmixing matrix [31, 32]. Extensive trials showed that the ICs which were computed through this dimension-reduction technique did not include the short-duration wavelets mentioned above. Instead, the ICs had waveforms with spindle-like morphology, that is, waveforms of gradually increasing and then decreasing amplitude, lasting for at least 0.5 second. 

### 2.3. Extraction of Spindle Components

The next step in the analysis consisted of dividing the original single-spindle timeframe into parts that reflected different spindle-like patterns, within that spindle. The rationale for such a temporal division is based on the observation that, within a single spindle, different spindle-like patterns may appear sequentially. The division was based on the existence, in the EEG recordings, of distinct waxing-waning cycles and/or on the existence of sustained transitions in instantaneous spindle frequency from “low” (≤12 Hz) to “high” (≥13 Hz) frequencies (or vice versa). The division was done manually on the bandpass-filtered EEG recordings, before application of ICA. The “division” point was located at the middle of the transition either from one waxing-waning cycle to the next one or from a low to a high (or vice versa) instantaneous spindle frequency. Frequencies equal to or higher than 14 Hz were considered “high” and, together with the “borderline-high” frequency of 13 Hz, were considered as representing the fast spindle class. The 13 Hz frequency was included in the fast spindle class, since the “border” between the spectra of the two spindle classes has been shown to be in the 12-13 Hz band [5, 33]. Accordingly, frequencies equal to or lower than 12 Hz were considered “low”, representing the slow spindle class.

After the division of the single-spindle timeframe into parts according to the decision process stated above, an inspection of the computed ICs followed. The aim was to select those ICs which possessed spindle-like morphology and would best correspond to the previously selected parts of the single-spindle timeframe, as far as their time duration and their frequency content were concerned. For each EEG part, some ICs were grouped together and were considered as “representative” (main) ICs for that part, according to the following procedure: For ICs to be considered as representative of a spindle part, they should have had spindle-like waveform extending to at least two-thirds of the entire time length of the EEG part. Additionally, their maximum-power frequency should have been in the same spindle frequency class (slow or fast) as that of the EEG in that part. However, even if an IC's spindle-like activity possessed temporal coincidence and had similar frequency content with an EEG part, it was not included in the representative IC group for that part if its spindle-like waveform extended significantly (i.e., for more than 0.5 second) into another part.

It should be noted that in the process of the approximate matching of the EEG parts to the spindle-like waveforms of the ICs, the initially chosen boundaries defining the EEG parts under examination could be modified. This modification was based on the information provided by the morphology of the ICs, because ICs could possess a much clearer starting and/or stopping point for the signal activity than the original filtered EEG.

After ICs had been selected as representative of the parts, the EEG was reconstructed, for the whole time duration of the spindle, based on the representative (main) ICs of each part. This led to the extraction, in the reconstructed EEG, of spindle components (SCs) corresponding to separate EEG activity patterns within the same spindle. 

### 2.4. Computation of Intracranial Current Sources

Numerous LORETA studies have used 19 or 21 electrodes of the 10/20 system [34–38]. Based on the results of these previous studies, the use of 21 electrodes in the present study was expected to provide acceptable localisation accuracy, despite the inevitable diffuseness in the current source locations produced by the method. However, we proceeded into an investigation of the lower limits of electrode use in LORETA-based inversions. Simulations were performed in order to check the extent to which the localization accuracy of the LORETA technique held, under the restriction of 8 and 16 electrodes available for the inversion. As expected, the computed current source distributions were extended over wide regions. Nevertheless, for the 16-electrode configuration, the positions of the local maxima of the computed source distributions, in conjunction with the topography of the surrounding “slopes”, corresponded to the correct current source locations.

The intracranial current sources were computed using LORETA, for each time sample, for both the original bandpass-filtered EEGs and the reconstructed ones. Accordingly, 3D distributions of source current density were estimated at the 2394 cortical locations utilized in LORETA [15]. The amount of information that was present in the current density signal sets, for all the time samples, was overwhelming and did not help in easily extracting information about the source distributions that corresponded to the original EEGs and the SCs. In order to extract such information in a concise manner, while obtaining an average measure of the magnitude of the current source density at each source region, the temporal mean of the current density amplitude was computed for the whole duration of the respective spindle part, for each of the 2394 cortical locations. It was expected that these mean current density maps would represent faithfully the most active cortical regions, on the average, for the respective duration of each spindle part.

## 3. Results

### 3.1. Spindle Components

From the set of sleep spindles available for processing, special attention was paid to spindles which possessed a spectral bimodality, that is, exhibiting both slow and fast SCs. Figure 1 shows the multichannel recordings of such a spindle. The spindle started as a high-frequency one, with main frequency at 13-14 Hz, and then, at almost all electrodes, a transition to low frequencies took place, starting at 12 Hz and then moving to lower frequencies (10-11 Hz). Based on the visual inspection of the recordings, the activity at the majority of the electrodes presented three distinct parts or waxing-waning cycles, suggesting the existence of 3 SCs, termed hereafter SC1, SC2, and SC3. The approximate durations of the cycles were 0–0.675 second, 0.675–1.375 second, and 1.375–2.3 second, respectively. The 1st cycle had a main frequency range of 13-14 Hz. The 2nd cycle presented an “intercycle” transition in many electrodes from 13 to 12 Hz. The 3rd cycle possessed a clear low-frequency content (10-11 Hz). 

**Figure 2:**
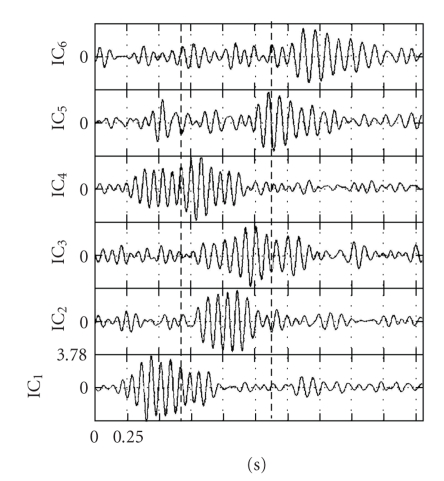
Independent Components (ICs) for the data in Figure 1. The exact IC values do not possess an interest, due to the sign and multiplicative constant indeterminacy of the results of ICA. The absolute maximum value of all ICs dictated a common magnitude range for the representation of the ICs. In each IC plot, the first (second) vertical dashed line indicates the time point of transition from part A (B) of the original single-spindle timeframe to part B (C).

The dimensionality reduction process resulted in computing *n*_red_ = 6 ICs, presented in Figure 2. Each IC possessed a visually discernible “main” waxing-waning cycle. By inspecting the starting and ending time of those cycles and their spectral content, and following the IC selection procedure described in Section 2.3, IC1, with main (i.e., maximum-power) frequency of its spindle-like waveform at 13 Hz, was selected as representative of part A and SC1. Its spindle-like waveform covered two-thirds of part A and its extension into part B was less than 0.5 second.

**Figure 3:**
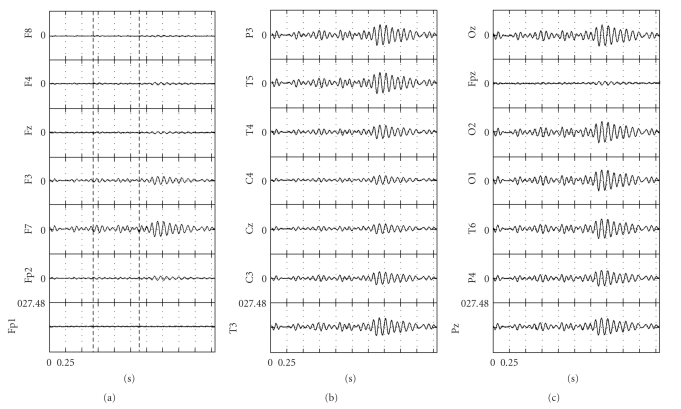
ICA-reconstructed EEG, based on IC6, corresponding to the SC (SC3), which is dominant in part C of the sleep spindle shown in Figure 1. In each electrode recording plot the first (second) vertical dashed line indicates the time point of transition from part A (B) of the original single-spindle timeframe to part B (C). Potentials of EEG are in microvolts.

IC2, with main frequency of its spindle-like waveform at 13 Hz, was selected as representative of part B and SC2, since its waveform covered almost the entire time length of part B and did not extend to either parts A or C. IC3, with main frequency of its spindle-like waveform at 12 Hz, was also selected as representative of part B and SC2, since its waveform covered almost the entire time length of part B and its extension into part C was less than 0.5 second. Finally, IC6, with main frequency of its spindle-like waveform at 11 Hz, was selected as representative of part C and SC3, since the waveform extended to more than two-thirds of part C and did not extend into part B. IC4 possessed a spindle-like waveform, with main frequencies at 13-14 Hz, spanning parts A and B. IC4 was not retained, because the waveform extended to 0.5 second in both parts A and B. IC5 possessed a spindle-like waveform, with main frequency at 12 Hz, spanning parts B and C. It was not included as a representative of either part B or C, since its spindle-like waveform did not extend to at least the two-thirds of the duration of part B or C. Figure 3 shows the reconstructed EEG, at 21 electrodes, based on IC6. The reconstructed EEG represents the spindle-like activity of SC3, which is the dominant SC in part C. In parts A and B, SC3 presented a much lower amplitude activity, which could hardly be characterized as spindle-like. Nevertheless, this activity could be a precursor to the clear emergence of SC3 in part C. 

### 3.2. Intracranial Current Sources

Concerning the mean current source activity corresponding to the original EEG data for the three parts into which the single-spindle EEG was segmented, in all three parts the maxima were located at the cuneus (occipital lobe) and at the temporal lobes, bilaterally (Table 1). A frontal distribution, with local maximum at the anterior cingulate, with intensity at 52 and 49% of the global maximum, appeared in the mean current source activity maps for parts B and C, respectively (Table 1 parts B and C). 

**Figure 4:**
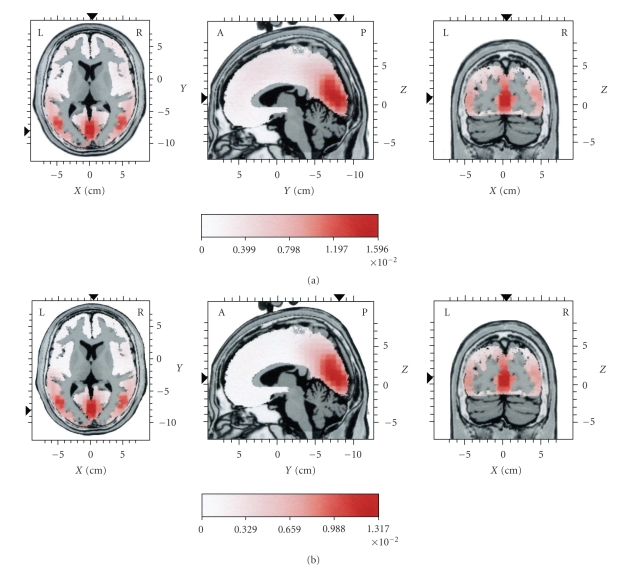
Distributions of mean current source activity for part A (see Figure 1). In (a) sources are given for the original EEG. In (b) sources are given for the reconstructed EEG, representing the dominant spindle component (SC1) in this part. The reconstruction of the EEG was based on IC1. Each distribution is displayed relative to its own maximum, using three slices (axial, sagittal, and coronal) intersecting at the point of global maximum of the distribution. Current density values are in *μ*A/mm^2^.

For part A, as mentioned above, the EEG frequency was high. Therefore, it was expected that the mean current source activity should appear as activation mainly at posterior parts (Figure 4(a)). It should be noted that, in the context of the present work, the term “posterior” is used mainly in contradistinction to frontal lobes, and it denotes current sources not only in the occipital lobes but also in the limbic, parietal, and temporal lobes. The mean current source activity map (Figure 4(b)) corresponding to SC1 (i.e., the reconstruction of the EEG based on IC1, for part A, where IC1 was the main IC) showed the same loci of maximal activity at the cuneus (occipital lobe) and at the temporal lobes, bilaterally (Table 2), as those present in the current sources corresponding to the original EEG for part A.

**Figure 5:**
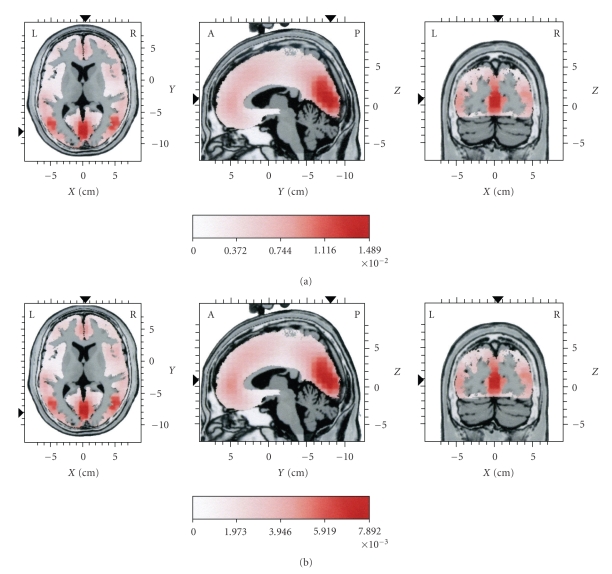
Distributions of mean current source activity for part B (see Figure 1). In (a) sources are given for the original EEG. In (b) sources are given for the reconstructed EEG, representing the dominant spindle component (SC2) in this part. The reconstruction of the EEG was based on IC2 and IC3. Each distribution is displayed relative to its own maximum, using three slices (axial, sagittal, and coronal) intersecting at the point of global maximum of the distribution. Current density values are in *μ*A/mm^2^.

For part B, the main current sources of the original EEG remained at the same posterior positions as for part A, namely, the cuneus and the middle temporal gyri, bilaterally (Figure 5(a) and Table 1 part B). However, a lower-intensity frontal component was also seen, at the anterior cingulate. As mentioned above, for part B the spindle frequency content remained high, although slower frequencies emerged. Therefore, the emergence of a lesser frontal source local maximum seems to be in agreement with the assumption that slow spindles tend to occur at frontal areas. The mean current source activity map (Figure 5(b)) corresponding to SC2 (i.e., the reconstruction of the EEG based on ICs 2 and 3, for part B, where ICs 2 and 3 were the main ICs) showed the same loci of maximal activity, at the cuneus, the temporal lobes, bilaterally, and the anterior cingulate (Table 3), as those present in the current sources corresponding to the original EEG for part B.

**Figure 6:**
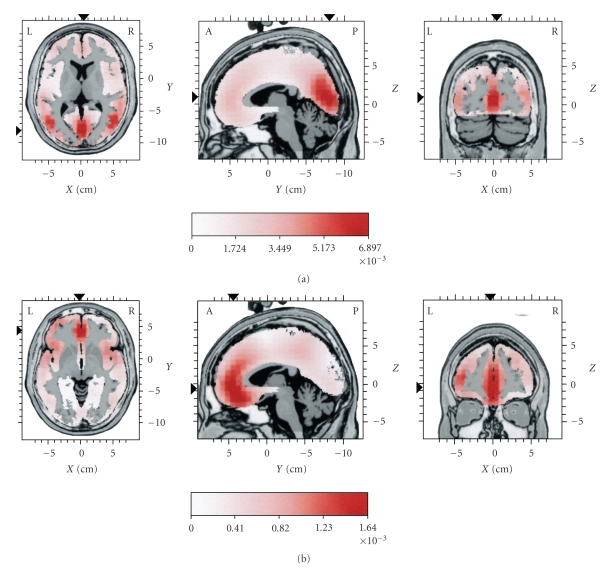
Distributions of mean current source activity for part C (see Figure 1). In (a) sources are given for the original EEG. In (b) sources are given for the reconstructed EEG, representing the dominant spindle component (SC3) in this part. The reconstruction of the EEG was based on IC6. Each distribution is displayed relative to its own maximum, using three slices (axial, sagittal, and coronal) intersecting at the point of global maximum of the distribution. Current density values are in *μ*A/mm^2^.

For part C, as mentioned above, the original EEG was of low frequency. Therefore, the observed dominance of posterior sources for part C of the original EEG (Figure 6(a) and Table 1 part C) did not agree with the assumption that slow spindles tend to occur at frontal areas. Figure 6(b) presents the mean current source activity map corresponding to SC3 (i.e., the reconstruction of the EEG based on IC6, for part C, where IC6 is the main IC). A clear frontal maximal distribution emerged, with lesser activity at temporal and parietal areas (Table 4). This was consistent with the low frequency content of the spindle-like activity that existed in the reconstructed EEG for part C.

It should be noted that the local maxima of the current source distribution for the reconstructed EEG based on IC6 were at the same spatial location for each part of the reconstructed EEG. However, the amplitude of the current density of the sources was increasing from part A to B and finally C, corresponding to the emergence of SC3 as the dominant spindle component of part C. Spatial stability for the local maxima of the current source distribution across the three parts of the EEG occurred also for SC1, that is, the EEG that was reconstructed based on IC1, and for SC2, that is, the EEG that was reconstructed based on IC2 and IC3.

## 4. Discussion

In the present study, the possibilities offered by ICA processing were explored for extracting sleep spindle components (SCs), in order to study the structure of sleep spindles during their temporal evolution. Observation of morphological characteristics of the obtained ICs and definition of distinct groups of ICs based on these characteristics proved quite helpful in elucidating such SCs. The results provide indication that SCs which relate to a single-spindle EEG recording, and which may not be easily distinguishable in the original recording, may be separated using morphological and frequency spectrum criteria, when these criteria are applied to the original single-spindle EEG recording and its ICs. The EEGs reconstructed from the different IC groups clearly indicated specific SCs active in consecutive parts of a single spindle. The sleep spindles were divided into consecutive time segments (parts) and at each segment the corresponding SC was found to provide the predominant spindle-like characteristics of the EEG. The above findings are in accordance to the feature of ICA processing related to the “unmixing” of the available recorded data into underlying components. 

One of the benefits of this approach was that the contribution of the current sources for each SC to the total EEG current source distribution could be differentiated, with interesting results concerning the sources of slow and fast spindle classes. In accordance with previous findings, we observed that fast SC activity related to activation of principally posterior brain parts, whereas slow SC activity related to activation of mainly anterior parts. On the other hand, there existed cases where the amplitude maxima of the total EEG current sources for some time parts of the original EEG were located posteriorly, although the dominant frequency of the original EEG, at those parts, was slow. 

A consistent finding in the spindles analysed in the present study was the spatial stationarity of the current sources for each SC, across consecutive reconstructed EEG parts of the same spindle. In conjunction with the results discussed in the previous paragraph, spatial stationarity of current sources provides an indication that slow and fast components of a single spindle (which may represent a frequency shift during its duration) may originate in different parts of the brain and reflect distinct groups of generators which remain active throughout the spindle duration. The intensity of these generators may be modulated in time, to reflect the changes in the frequency content of the spindle as a function of time.

Future research should include the application of the proposed technique of SC extraction and related current source estimation to a set of healthy young adults, and then to healthy subjects of all ages, in order to investigate possible age effects on SCs. Furthermore, taking into account the limitations of LORETA [13], current sources should be investigated with inversion techniques using different and/or more comprehensive methodological approaches than LORETA [39, 40], in order to check whether the present findings can be replicated with such techniques. An extensive investigation, from the point of view of inversion techniques, might also contribute in a more robust manner to the elucidation of the questions related to the existence and location of functionally separate sleep spindle generators, for the fast and slow spindle classes.

## Figures and Tables

**Table 1:** Local maxima of LORETA current source density distributions for the original EEG, for the three parts (A, B, and C, resp.) into which the single-spindle timeframe was divided. The local maxima greater than or equal to 50% of the global maximum are shown (49% for the part C subtable). Coordinates are in mm. Origin at anterior commisure. For *X*, negative values represent left; positive values represent right. For *Y*, negative values represent posterior; positive values represent anterior. For *Z*, negative values represent inferior; positive values represent superior. Brodmann areas (BA) and both descriptions of the anatomical regions are shown.

Part A							
Local maximum	Coordinates in Talairach space			Brodmann area (BA)	Anatomical region 1	Anatomical region 2	Activity (*10^−3^ ) (*μ*A/mm^2^)

	*X*	*Y*	*Z*				
1	4	−81	8	17	Cuneus	Occipital Lobe	15.96
2	−52	−67	8	39	Middle Temporal Gyrus	Temporal Lobe	13.14
3	−45	−67	15	39	Middle Temporal Gyrus	Temporal Lobe	13.14
4	46	−67	15	37	Middle Temporal Gyrus	Temporal Lobe	13.08

Part B							

Local maximum	Coordinates in Talairach space			Brodmann area (BA)	Anatomical region 1	Anatomical region 2	Activity (*10^−3^ ) (*μ*A/mm^2^)

	*X*	*Y*	*Z*				
1	4	−81	8	17	Cuneus	Occipital Lobe	14.88
2	46	−67	8	37	Middle Temporal Gyrus	Temporal Lobe	12.32
3	−45	−67	15	39	Middle Temporal Gyrus	Temporal Lobe	12.20
4	−3	52	1	10	Anterior Cingulate	Limbic Lobe	7.80
5	53	3	−13	21	Middle Temporal Gyrus	Temporal Lobe	7.76
6	−59	−32	8	42	Superior Temporal Gyrus	Temporal Lobe	7.71

Part C							

Local maximum	Coordinates in Talairach space			Brodmann area (BA)	Anatomical region 1	Anatomical region 2	Activity (*10^−3^ ) (*μ*A/mm^2^)

	*X*	*Y*	*Z*				
1	4	−81	8	17	Cuneus	Occipital Lobe	6.90
2	46	−67	8	37	Middle Temporal Gyrus	Temporal Lobe	6.17
3	−45	−67	15	39	Middle Temporal Gyrus	Temporal Lobe	6.00
4	−59	−32	8	42	Superior Temporal Gyrus	Temporal Lobe	3.52
5	−3	52	1	10	Anterior Cingulate	Limbic Lobe	3.44

**Table 2:** Local maxima of LORETA current source density distributions for spindle component SC1 for part A of the single-spindle timeframe. The local maxima greater than or equal to 50% of the global maximum are shown. Coordinates are in mm. Origin at anterior commisure. For *X*, negative values represent left; positive values represent right. For *Y*, negative values represent posterior, positive values represent anterior. For *Z*, negative values represent inferior, positive values represent superior. Brodmann areas (BA) and both descriptions of the anatomical regions are shown.

Local maximum	Coordinates in Talairach space			Brodmann area (BA)	Anatomical region 1	Anatomical region 2	Activity (*10^−3^) (*μ*A/mm^2^)
	*X*	*Y*	*Z*				
1	4	−81	8	17	Cuneus	Occipital Lobe	13.17
2	−52	−67	8	39	Middle Temporal Gyrus	Temporal Lobe	11.06
3	46	−67	8	37	Middle Temporal Gyrus	Temporal Lobe	10.49

**Table 3:** Local maxima of LORETA current source density distributions for spindle component SC2 for part B of the single-spindle timeframe. The local maxima greater than or equal to 50% of the global maximum are shown. Coordinates are in mm. Origin is at anterior commisure. For *X*, negative values represent left; positive values represent right. For *Y*, negative values represent posterior; positive values represent anterior. For *Z*, negative values represent inferior; positive values represent superior. Brodmann areas (BA) and both descriptions of the anatomical regions are shown.

Local maximum	Coordinates in Talairach space			Brodmann area (BA)	Anatomical region 1	Anatomical region 2	Activity (*10^−3^) (*μ*A/mm^2^)
	*X*	*Y*	*Z*				
1	4	−81	8	17	Cuneus	Occipital Lobe	7.89
2	46	−67	8	37	Middle Temporal Gyrus	Temporal Lobe	6.65
3	−45	−67	15	39	Middle Temporal Gyrus	Temporal Lobe	6.34
4	−3	52	1	10	Anterior Cingulate	Limbic Lobe	4.89
5	53	3	−13	21	Middle Temporal Gyrus	Temporal Lobe	4.83
6	−59	−32	8	42	Superior Temporal Gyrus	Temporal Lobe	4.39

**Table 4:** Local maxima of LORETA current source density distributions for spindle component SC3 for part C of the single-spindle timeframe. The local maxima greater than or equal to 50% of the global maximum are shown. Coordinates are in mm. Origin is at anterior commisure. For *X*, negative values represent left; positive values represent right. For *Y*, negative values represent posterior; positive values represent anterior. For *Z*, negative values represent inferior; positive values represent superior. Brodmann areas (BA) and both descriptions of the anatomical regions are shown.

Local maximum	Coordinates in Talairach space			Brodmann area (BA)	Anatomical region 1	Anatomical region 2	Activity (*10^−3^) (*μ*A/mm^2^)
	*X*	*Y*	*Z*				
1	−3	45	6	10	Medial Frontal Gyrus	Frontal Lobe	1.64
2	−38	52	8	10	Medial Frontal Gyrus	Frontal Lobe	1.45
3	46	10	36	9	Medial Frontal Gyrus	Frontal Lobe	1.41
4	−52	3	−20	21	Middle Temporal Gyrus	Temporal Lobe	1.39
5	53	10	8	44	Inferior Frontal Gyrus	Frontal Lobe	1.35
6	46	10	1	13	Insula	Sub-lobar	1.35
7	−38	17	1	13	Insula	Sub-lobar	1.23
8	−45	−46	50	40	Inferior Parietal Lobule	Parietal Lobe	1.11
9	−52	−60	36	40	Inferior Parietal Lobule	Parietal Lobe	1.00
10	−52	−60	15	22	Superior Temporal Gyrus	Temporal Lobe	0.89
11	32	45	29	10	Medial Frontal Gyrus	Frontal Lobe	0.87
12	−59	−32	22	40	Inferior Parietal Lobule	Parietal Lobe	0.86
13	25	−4	−27	Amygdala	Uncus	Limbic Lobe	0.86
14	25	38	43	8	Superior Frontal Gyrus	Frontal Lobe	0.84
